# Aldosterone Disrupts the Intercellular Flow of Glucose in Cardiac Muscle

**DOI:** 10.3389/fendo.2015.00185

**Published:** 2015-12-11

**Authors:** Walmor C. De Mello

**Affiliations:** ^1^School of Medicine, University of Puerto Rico, San Juan, PR, USA

**Keywords:** aldosterone, gap junction permeability, glucose heart

## Abstract

The activation of the renin–angiotensin system is known to impair intercellular communication in the heart, but the role of aldosterone on the process of chemical communication and particularly the intercellular diffusion of glucose between cardiomyocytes is not known. This problem was investigated in cell pairs isolated from the left ventricle of adult Wistar Kyoto rats. For this, fluorescent glucose was dialyzed into one cell of the pair using the whole cell clamp technique, and its diffusion from cell-to-cell through gap junctions was followed by measuring the fluorescence intensity in the dialyzed as well as in non-dialyzed cell as a function of time. The results indicated that (1) in cell pairs exposed to aldosterone (100 nM) for 24 h, the intercellular flow of glucose through gap junctions was disrupted; (2) although the mechanism by which aldosterone disrupts the cell-to-cell flow of glucose is multifactorial, two major factors are involved: oxidative stress and PKC activation; (3) the effect of aldosterone was significantly reduced by spironolactone (100 nM); and (4) calculation of gap junction permeability (Pj) indicated an average values of 0.3 ± 0.001 × 10^−4^ cm/s (*n* = 31) (four animals) for controls and 24 ± 0.03 × 10^−6^ cm/s (*n* = 34) (four animals) (*P* < 0.05) for cell pairs exposed to aldosterone (100 nM) for 24 h. Bis-1 (10^−9^M), which is a selective PKC inhibitor, added to the aldosterone solution, improved the value of Pj to 0.21 ± 0.001 × 10^−4^ cm/s (*n* = 24) (*P* < 0.05), whereas spironolactone (100 nM) added to aldosterone solution, reduced significantly the effect of the hormone on junctional permeability to glucose.

## Highlights

- Aldosterone inhibits the cell-to-cell diffusion of glucose in heart.
- Spironolactone inhibits the effect of aldosterone.
- The gap junction permeability is drastically reduced by aldosterone.
- Oxidative stress is involved in the effect of aldosterone.

## Introduction

Aldosterone is normally involved in the regulation of blood volume and blood pressure. In the last decades, evidence has been provided that the hormone has many effects independently of its regulatory role on blood volume. Aldosterone, for instance, binds to the mineralocorticoid receptor (MR), which resides predominantly in the cytoplasm. Upon ligand binding, the MR acts as a transcription factor generating cardiac remodeling, including hypertrophy and fibrosis (1–5), especially during heart failure – an effect blocked by spironolactone (6). Moreover, the hormone enhances the expression of Ang II AT1 receptors in ventricular muscle by twofold (3), an effect inhibited by eplerenone (4), and it is involved in a proliferative response through increased expression of p53-bing protein (7). During pathological conditions, such as myocardial infarction, the production of aldosterone in the cardiac muscle is enhanced, (5) and the cardiac levels of Ang II are increased (7). Both aldosterone and Ang II are involved in the increase of collagen deposition during myocardial infarction [see Ref. (8)], creating a severe impairment of ventricular relaxation and impulse propagation with generation of cardiac arrhythmias. Transcription effects of aldosterone take more than 3 h, whereas rapid non-genomic effects of the hormone can occurs within minutes. Signaling pathways related to the rapid MR/aldosterone responses include the MAPK family and protein kinase C (PKC) (9) as well as intracellular levels of calcium, cAMP, and nitric oxide (10, 11).

Clinical trials involving patients with heart failure revealed that MR antagonists reduces morbidity and mortality (6) even when the circulating levels of aldosterone are within normal range, highly suggesting that the activation of local renin–angiotensin aldosterone system (RAAS) is an important contributor factor during heart failure. Other clinical studies showed that elevated plasma aldosterone levels facilitate the development of insulin resistance by increasing oxidative stress and by changing insulin signaling with consequent decrease of glucose transport. Furthermore, enhanced aldosterone levels are associated with myocardial infarction and stroke (12).

Cardiac cells are communicated through gap junction channels which are composed of two oligomers and each oligomer is a connexon which contains connexins (13). Connexin43 (Cx43) is the main connexin present in cardiac muscle. Connexins contain four transmembrane domains with the amino and carboxyl termini on the cytoplasmic side. The intercellular channels make possible the spread of ions and electrical current from cell-to-cell, which is essential for the electrical synchronization in the heart (14), and is also involved in the intercellular diffusion of amino acids, nucleotides, hormones, and other small molecules up to 1 kDa contributing to the metabolic cooperation between cells (15, 16). Aldosterone reduces the expression of connexin43 (Cx43), which is the main connexin in cardiac muscle (17). Recently, it was found that glucose flows from cell-to-cell through gap junctions in cardiac muscle (16), which indicates that cardiomyocytes share this energy substrate and cooperate metabolically (16, 18). No information is available if aldosterone influences the intercellular diffusion of glucose. These observations might have important implications for heart failure, hypertension, and diabetes when the RAAS is activated. In this work, this problem was investigated in ventricular myocytes isolated from adult Wistar Kyoto rats.

## Materials and Methods

Normal adult Wistar Kyoto rats were used. The animals were kept in the Animal House at constant temperature (24°C) and humidity following the recommendations of NIH. Animals were kept on a normal laboratory animal diet and given tap water *ad libitum*. The animals were anesthetized with 43 mg/kg of ketamine plus 5 mg/kg of xylazine, and the heart was removed with the animals under deep anesthesia. All animal procedures were approved by the IACUC.

### Cell Isolation Procedure

The heart was removed and immediately perfused with normal Krebs solution containing (millimolar): NaCl – 136.5; KCl – 5.4; CaCl_2_ – 1.8; MgCl_2_ – 0.53; NaH_2_PO_4_ – 0.3; NaHCO_3_ – 11.9; glucose – 5.5; HEPES – 5, pH adjusted to 7.3. After 20 min, a Ca-free solution containing 0.4% collagenase (Worthington Biochemical Corp.) was recirculated through the heart for 1 h. The collagenase solution was washed out with 100 ml of recovery solution containing (millimolar): taurine 10, oxalic acid 10, glutamic acid 70, KCl 25, KH_2_PO_4_ 10, glucose 10, and EGTA 0.5; pH 7.4. All solutions were oxygenated with 100% O_2_. Ventricles were minced (1–2 mm thick slices) and the resulting solution was agitated gently and the suspension was filtered. The filtrate was centrifuged for 4 min at 22 × *g*. The cell pellets were then resuspended in normal Krebs solution.

### Experimental Procedures

All experiments were performed in a small chamber mounted on the stage of an inverted phase-contrast microscope (Diaphot, Nikon). Ventricular cells were placed in a modified cultured dish (volume 0.75 ml) in an open-perfusion microincubator (Model PDMI-2, Medical Systems). Cells were allowed to adhere to the bottom of the chamber for 15 min and were superfused with normal Krebs solution (3 ml/min) that permits a complete change of the bath in <500 ms. A video system made possible to inspect the cells and the pipettes throughout the experiments. The electrical measurements were carried out using the patch clamp technique in a whole cell configuration using an Axon (model 200B) patch-clamp amplifier and Digidata 1400 (Molecular Devices, CA, USA).

### Measurements of Intercellular Diffusion of Glucose

Cell pairs of ventricular myocytes were used. Suction pipettes were pulled from microhematocrit tubing by means of a controlled puller (Narashige, Japan) and filled with a solution with the following composition (millimolar): cesium aspartate 120, NaCl 10, MgCl_2_ 3, EGTA 10, tetraethylammonium chloride 20, Na_2_ATP 5, and HEPES 5; pH 7.3. In some experiments, fluorescent glucose (molecular weight 180 Da) (4%) was added to the pipettes. The pipette was attached to one cell of the pair, a gigaohm seal was achieved and then the membrane was ruptured by a brief suction allowing the fluorescent glucose to diffuse from the pipette into the cell.

### Measurements of Cell Width

Measurements of cell width – a major component of cardiac cell volume variation – were made in quiescent ventricular myocytes incubated for 24 h with Krebs solution containing aldosterone (100 nM), an inverted phase-contrast microscope (Nikon), and a high-resolution camera (Paxcam). Cell width was measured using Image J software (150 randomly chosen cells/well, NIH, Bethesda, MD, USA).

### Measurements of Oxidative Stress

To directly monitor real-time reactive oxygen/nitrogen species (ROS/RNS), a kit including an oxidative stress detection reagent (ENZO Life Sciences, Farmingdale, NY, USA) was used. Measurements of fluorescence intensity were made in controls and in cells exposed to aldosterone (100 nM) for 24 h. The oxygen/nitrogen species kit was added for 30 min and the green fluorescence was measured again using a wide filed fluorescence microscope equipped with standard green (490/525 nm) filter set.

### Influence of PKC Inhibition

The influence of PKC activation on the effect of aldosterone was investigated. For this, cell pairs were exposed to aldosterone (100 nM) plus Bis-1 (10^−9^M), which is a selective inhibitor of PKC. After 24 h, fluorescent glucose was dialyzed into cell pairs as described above and cell-to-cell diffusion of glucose was followed as a function of time.

### Drugs

Aldosterone and spironolactone were from Sigma Chemical Co., Saint Louis, MO, USA. Bis-1 was from Calbiochem.

### Statistical Analysis

Data are expressed as mean ± SEM. Student’s *t*-test was used. Differences were considered significant when *P* < 0.05.

## Results

### Aldosterone Disrupts Intercellular Flow of Glucose

**Figure 1:**
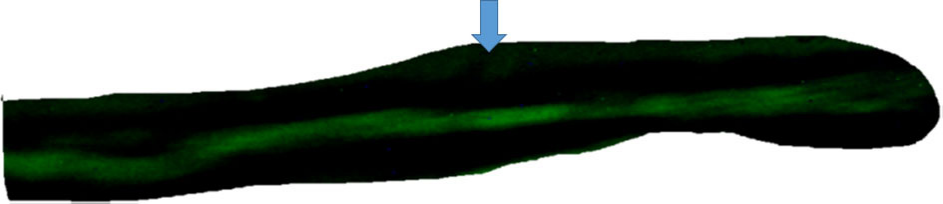
**Top – absence of intercellular diffusion of fluorescent glucose in single cell pair exposed to aldosterone (100 nM) for 24 h**. Arrow indicates site of micropipette filled with glucose.

**Figure 2:**
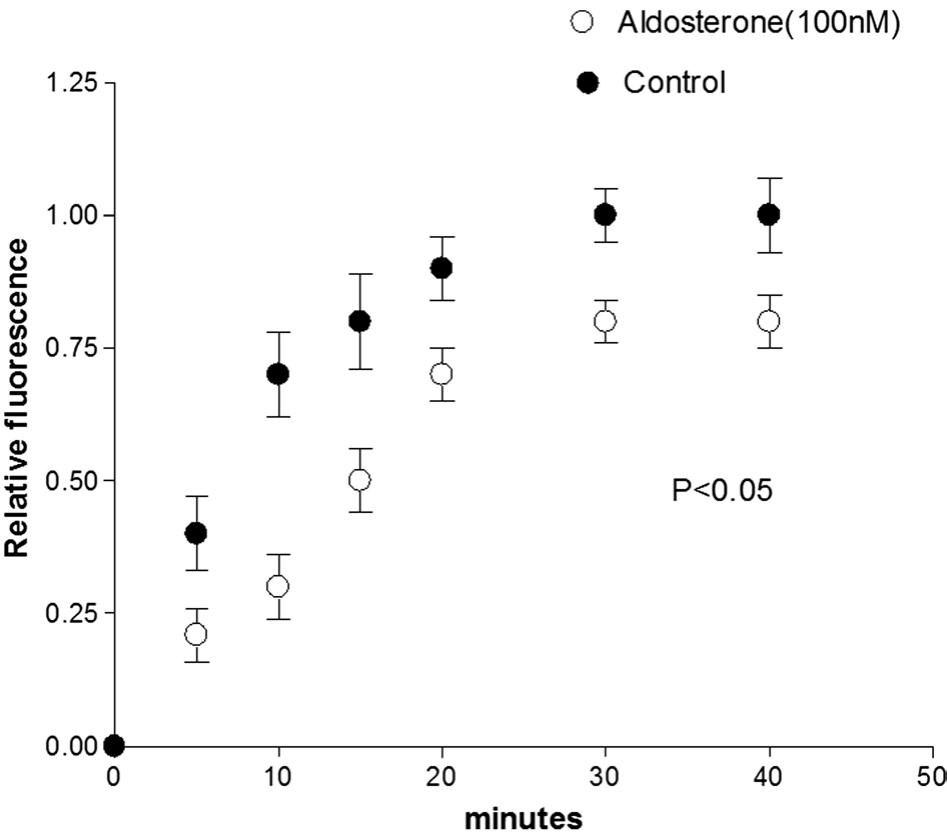
**Inhibition of cell-to-cell diffusion of fluorescent glucose in cell pairs exposed to aldosterone (100 nM) for 24 h**. Each point is the average from 25 cell pairs (four animals) isolated from the left ventricle. Vertical line at each point SEM (*P* < 0.05).

Previous observations indicated that in the normal heart, glucose flows from cell-to-cell through gap junctions (16). To investigate the influence of aldosterone on the intercellular diffusion of glucose, cell pairs isolated from the left ventricle of normal Wistar Kyoto rats were used. Fluorescent glucose was dialyzed into one cell of the pair and its diffusion through the gap junction was followed by measuring the fluorescence intensity in both cells of the pair as a function of time. As shown in Figures 1 and 2, in cell pairs exposed to aldosterone (100 nM) for 24 h, the intercellular movement of glucose was drastically reduced. Exposure of the cell pairs to aldosterone (100 nM) for 15 min did not change the junctional permeability to glucose (not shown).

**Figure 3:**
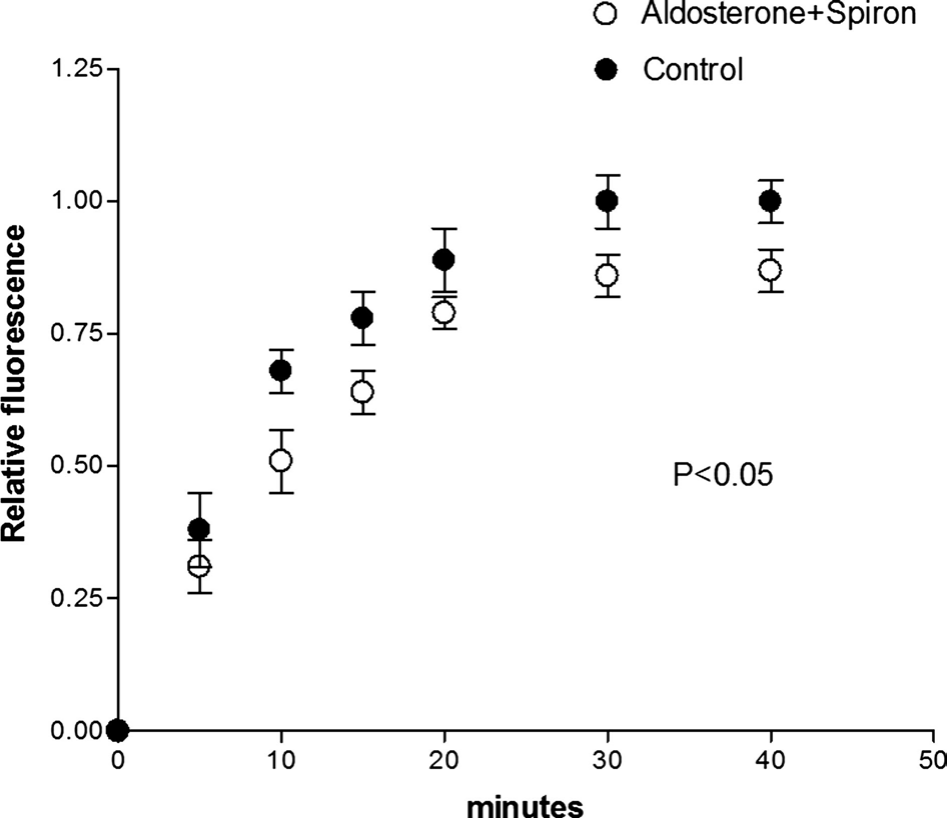
**Spironolactone (100 nM) added to aldosterone (100 nM) solution reduced the effect of aldosterone on the intercellular flow of glucose**. Each point is the average from 26 cell pairs. Vertical line at each point SEM (*P* < 0.05).

To investigate the role of the MRs on the effect of aldosterone, cell pairs were exposed to aldosterone (100 nM) plus spironolactone (100 nM) for a period of 24 h. Measurements of intercellular diffusion of glucose performed at the end of this time indicated that spironolactone reduces the effect of aldosterone on the intercellular flow of glucose as shown in Figure 3.

### Effect of Aldosterone on Cell Width

**Figure 4:**
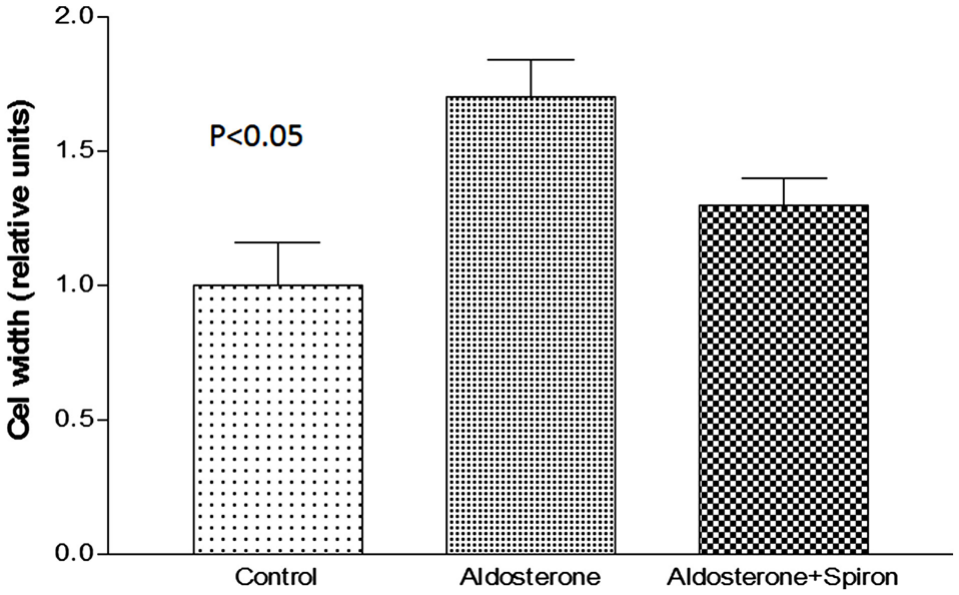
**Increase of cell width elicited by aldosterone (100 nM) and the effect of spironolactone (100 nM) on cell swelling induced by aldosterone**. Each bar is the average from 20 cells. Vertical line at each bar SEM (*P* < 0.05).

Because cell swelling is known to cause a decline of gap junction permeability (19), it is important to investigate if the hormone changes the cell volume of cardiac cells. To investigate this possibility, measurements of cell width were performed in absence and after 24 h of exposure to aldosterone (100 nM). Figure 4 shows that aldosterone increases the cell width significantly, while spironolactone (100 nM) added to aldosterone solution reduced significantly the effect of the hormone on cell width.

### Role of Oxidative Stress

**Figure 5:**
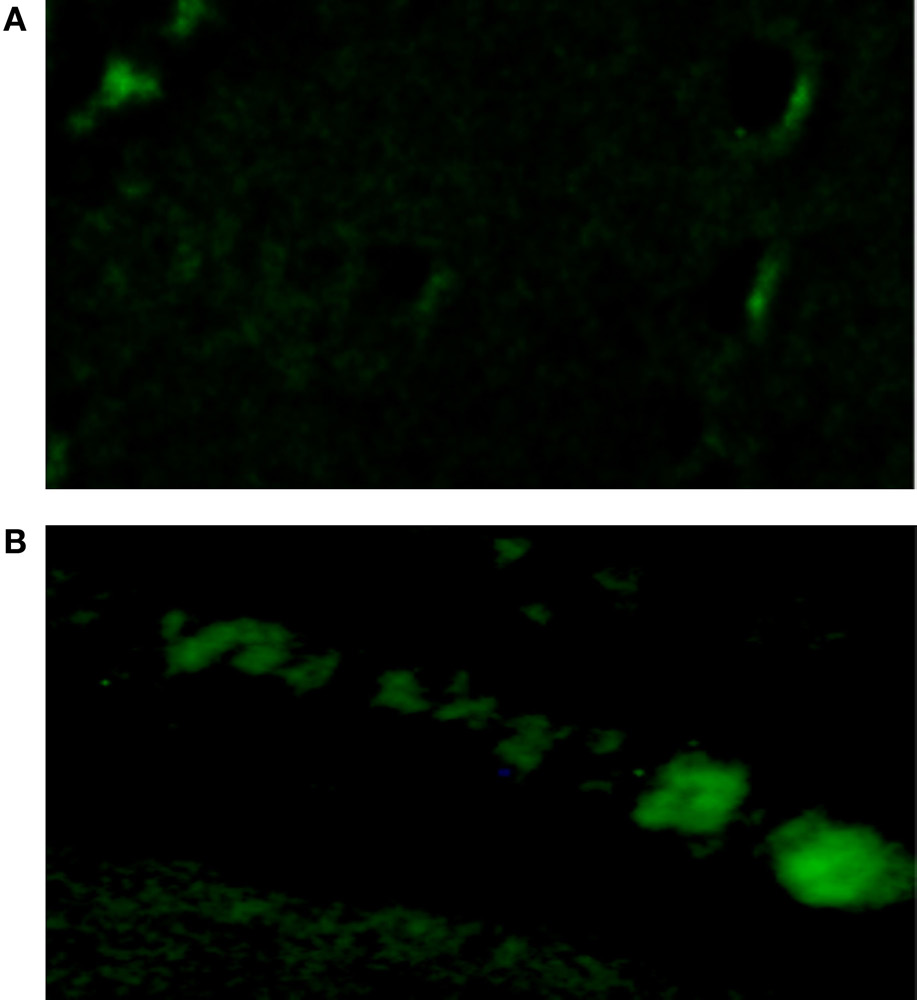
**Influence of aldosterone on oxidative stress**. **(A)** Control fluorescence microscopy of single cell under control conditions, left bottom; **(B)** green fluorescence measured in the same cell after administration of aldosterone (100 nM).

**Figure 6:**
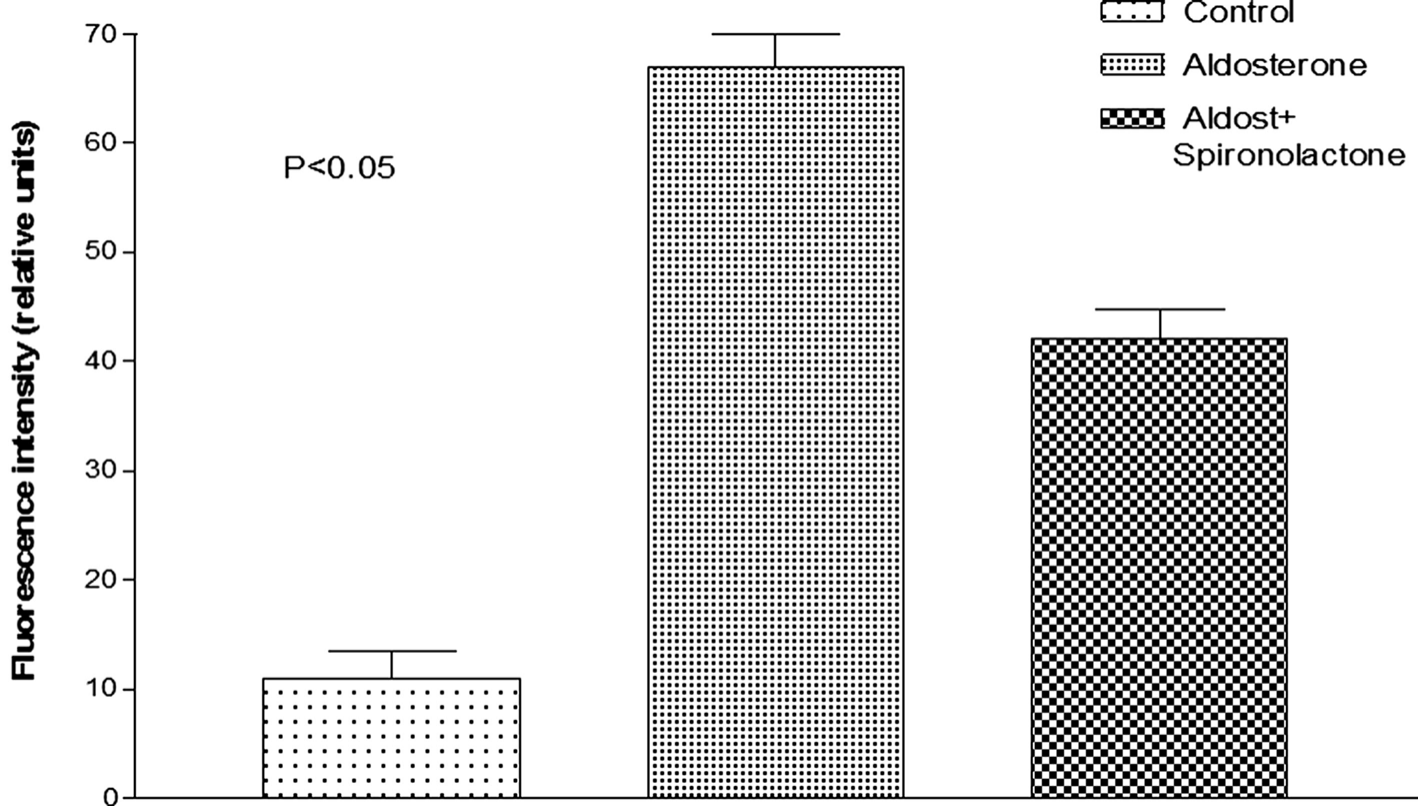
**Influence of aldosterone on oxidative stress**. Each bar is the average from 23 ventricular myocytes (4 animals). Vertical line at each bar SEM (*P* < 0.05).

Oxidative stress induced by the activation of MR is known to cause diastolic dysfunction and ventricular hypertrophy in rats (20). To investigate the possible role of oxidative stress on the decoupling action of aldosterone, isolated cells were incubated with Krebs solution containing aldosterone (100 nM) for 24 h and then exposed to the oxidative stress detection reagent for 30 min. Measurements revealed that the green fluorescence intensity inside the cells was significantly increased in cells incubated with aldosterone when compared with controls (see Figures 5 and 6).

In some experiments, measurements of reactive species were performed in presence of an inhibitor of ROS/RNS (ENZO Life Sciences). The results revealed a significant decline of green fluorescence elicited by aldosterone (100 nM). Moreover, measurements of gap junction permeability to glucose performed under these conditions indicated larger values of junctional permeability, Pj = 0.1 ± 0.002 × 10^−4^ cm/s (*n* = 12) (*P* < 0.05).

### Role of PKC Inhibition

**Figure 7:**
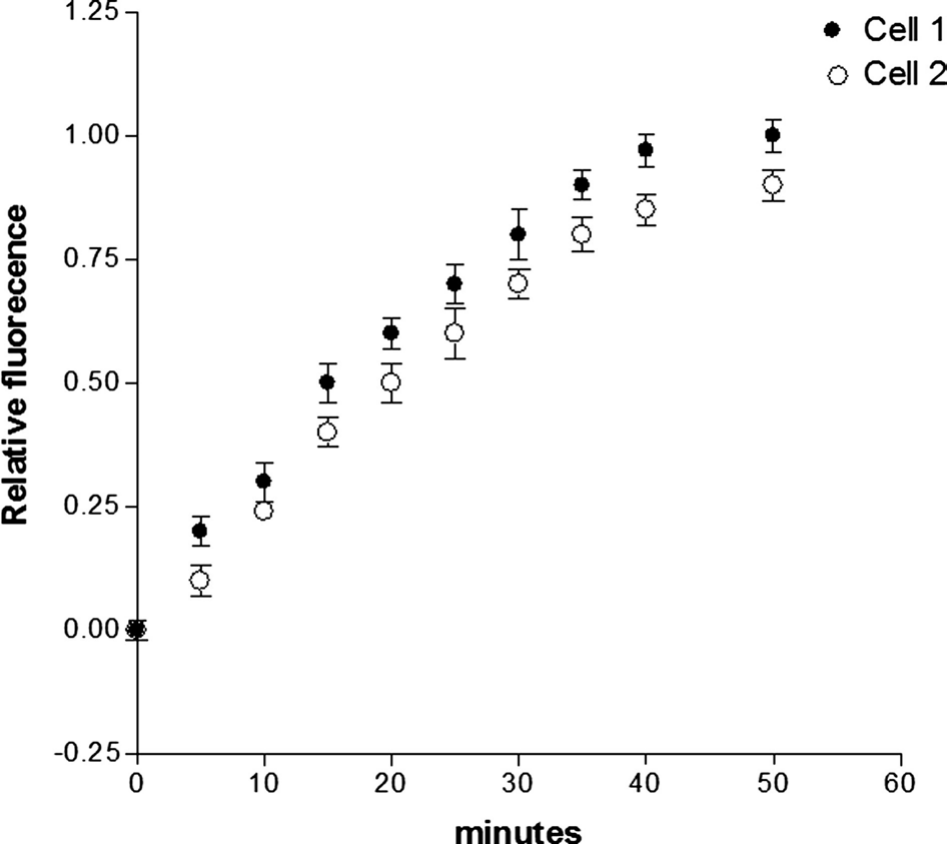
**Influence of PKC inhibition on the effect of aldosterone on junctional permability to glucose**. Cell pairs were exposed to aldosterone (100 nM) plus Bis-1 (10^−9^M) for 24 h and cell-to-cell diffusion of glucose was investigated. Each point is the average from 23 cell pairs (3 animals). Vertical line at each point SEM (*P* < 0.05).

Previous findings revealed that activation of PKC and consequent phosphorylation of connexin 43 decreases the dye coupling between cells increasing the preponderance of lower values of single channel conductance and permeability (21, 22). These observations led us to investigate if aldosterone reduces the gap junction permeability to glucose by activating PKC. For this, Bis-1, which is an inhibitor of PKC (10^−9^M), was added to aldosterone solution and after 24 h fluorescent glucose was dialyzed into the cell, and its intercellular diffusion was followed as a function of time. The results from several experiments indicated that the PKC inhibitor reduces drastically the effect of aldosterone on junctional permability (see Figure 7).

### Quantitative Measurements of Gap Junction Permeability

A quantitative estimation of the gap junction permeability to glucose (Pj) was made using the following equation (23):

Pj=Vcell/Aj×Kj

where Vcell is the cell volume that is accessible to glucose, Aj is area of the gap junctional membrane, and Kj is the rate constant of the transjunctional diffusion (23). Morphometric studies performed on ventricular tissues (24) indicate that Aj contributes to 17% of the cross sectional area of the rat cardiomyocyte (177 mm^2^) and the myofibrils, mitochondria, sarcoplasmic reticulum, and nucleus occupies about 88% of the total cell volume leaving 12% of total cell volume free for the diffusion of glucose through the cytoplasm. The junctional permeability to glucose (Pj) was then calculated for controls and for cell pairs exposed to aldosterone (100 nM) or to aldosterone (100 nM) plus spironolactone (100 nM) for 24 h. As shown in Table 1, Pj calculated for the controls taken Vcell as 12% of total cell volume was equal to 0.3 ± 0.001 × 10^−4^ cm/s (*n* = 31) (four animals). An appreciable decrease of junctional permeability was found in cell pairs exposed to aldosterone (100 nM) [24 ± 0.03 × 10^−6^ cm/s (*n* = 34) (four animals) (*P* < 0.05)] taking 12% of the total volume, which was assumed 60% larger than the controls due to cell swelling (see Table 1). In cell pairs exposed to aldosterone plus spironolactone (100 nM), the value of Pj was significantly greater than that achieved with aldosterone alone (see Table 1). The value of junctional permeability (Pj) in presence of Bis-1 (10^−9^M) and aldosterone (100 nM) was 0.21 ± 0.0011 × 10^−4^ cm/s (*n* = 24) (*P* < 0.05).

**Table 1:** **Influence of aldosterone alone and aldosterone (100 nM) plus spironolactone (100 nM) on the gap junction permeability (Pj) (cm/s) to glucose**.

Control	Aldosterone	Aldosterone plus spironolactone
0.3 ± 0.001 × 10^−4^ (*n* = 31) (4 animals)	24 ± 0.003 × 10^−6^ (*n* = 34) (4 animals)	2.4 ± 0.004 × 10^−5^ (*n* = 29) (4 animals)
	*P* < 0.05	*P* < 0.05

*Cells were exposed to aldosterone or aldosterone plus spironolactone for 24 h*.

## Discussion

The present results indicated, for the first time, that aldosterone disrupts chemical communication and the intercellular diffusion of glucose in the normal heart – an effect related to the activation of the MR. Since Ang II is known to release aldosterone by the adrenal gland, it is possible to conclude that the activation of the RAAS is involved in the regulation of intercellular diffusion of glucose in the heart. The mechanism by which the MR activation disrupts the cell-to-cell diffusion of glucose seems complex and multifactorial (see Figure 5) mainly involving the generation of oxidative stress, the downregulation of connexin43 (Cx43) (17), which is the main connexin in the heart and PKC activation. Aldosterone might contribute to the deterioration of chemical coupling by activation ERK, JNK, and p38 MAPKs (25). On the other hand, the effect of the hormone on the intracellular ion regulation (26) cannot be discarded. It is known, for instance, that aldosterone, rapidly increases [Na^+^]i and cell volume via Na^+^ K^+^ 2Cl^−^ cotransporter (NKCCl) and Na^+^/H^+^ exchange (NHE) (26). An increment of cell width was indeed, found in cells exposed to aldosterone for 24 h (see above). Since cell swelling decreases the gap junction permeability (19), it is conceivable that the cell swelling induced by aldosterone contributes to the disruption of cell-to-cell diffusion of glucose as described above.

Concerning the possible effect of aldosterone on the expression of Cx43, previous observations revealed that the downregulation of Cx43 expression elicited by aldosterone was not suppressed by eplerenone (17). Other studies indicated that spironolactone reverses gap junction remodeling during ventricular hypertrophy (27). Since spirinolactone decreased significantly the effect of aldosterone on the intercellular flow of glucose, the contribution of a decline in Cx43 remodeling to the disruption of intercellular movement of glucose cannot be ruled out.

**Figure 8:**
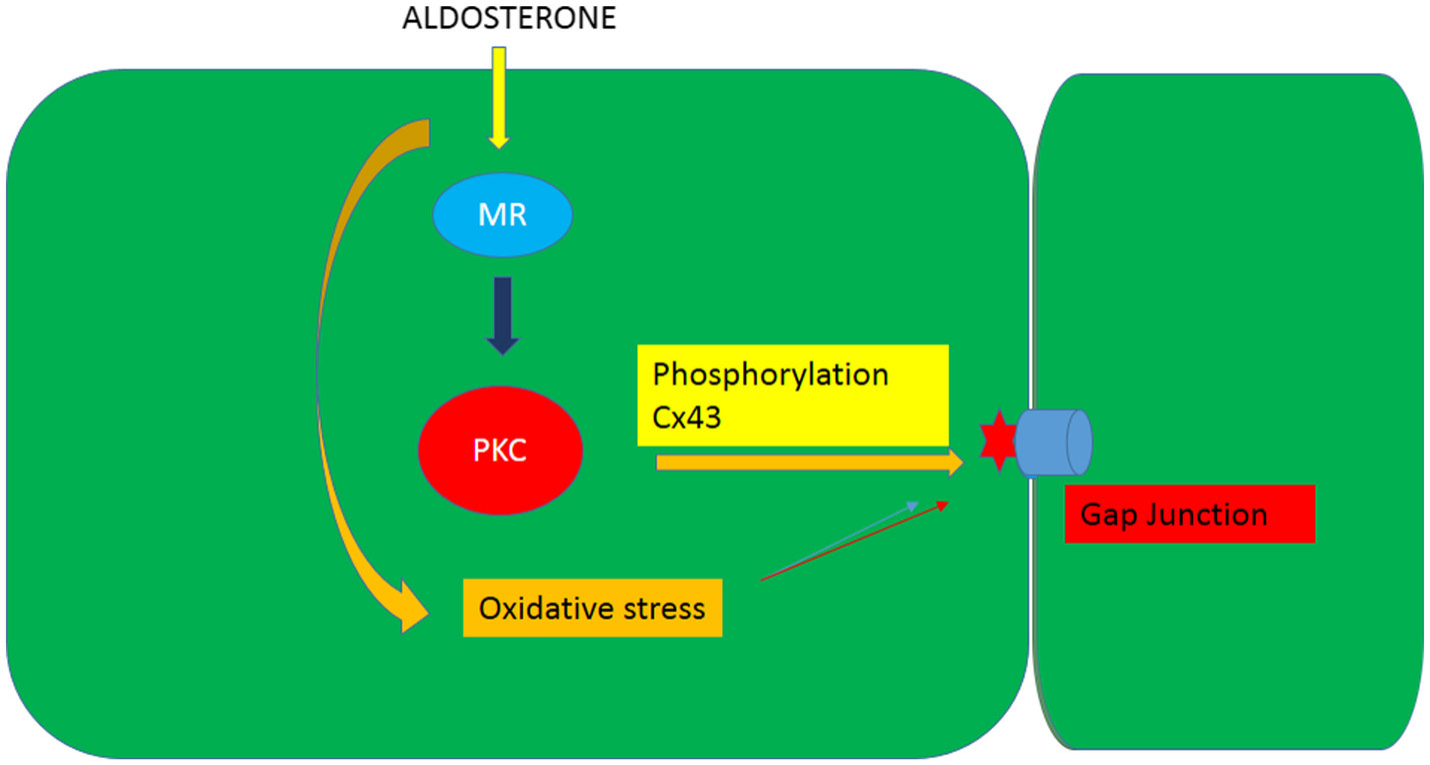
**Diagram illustrating the influence of aldosterone on the junctional permeability (Pj) to glucose through the generation of oxidative stress an activation of PKC**.

It is known that phosphorylation of gap junction proteins including connexin43, which is the main connexin in cardiac muscle, leads to a decrease of gap junction permeability and conductance (21, 22). On the other hand, evidence is available that aldosterone induces cellular effects through activation of PKC [see Ref. (28)] and that PKC activation may result from oxidative stress or lead to ROS generation. Indeed, PKC-dependent phosphorylation of Ser368 in Cx43 decreases intercellular coupling due to altered junctional permeability (29). These findings might indicate that the decline of intercellular flow of glucose caused by aldosterone is related to oxidative stress-induced PKC activation as shown in Figures 7 and 8.

Although it is well established that aldosterone produces MR-mediated injury, the hormone mediates part of Ang II-induced cardiac injury and gene expression suggesting a cross-talk between the hormone and the peptide. Previous studies showed that high glucose impairs the intercellular flow of glucose due, in part, due to the increased Ang II levels inside the cells (16, 18). Moreover, the enhanced synthesis of aldosterone during myocardial infarction is mediated by Ang II AT1 receptors (3, 30), and beta arrestin generated locally plays a role in Ang II-mediated aldosterone production (31). It is then reasonable to think that Ang II generated by the activation of the RAS can contribute to the decoupling action of aldosterone as described above by activating PKC and inducing oxidative stress.

It is known that not only aldosterone but also cortisol can cause cell death during MI (32, 33) indicating an important role of glucorticoids. Since no studies were included on the influence of cortisol on the intercellular diffusion of glucose, further observations will be needed to clarify this point.

Previous observations showed that mitochondria oxidative stress contributes to Ang II-induced gap junction remodeling (34) and that mitochondria can generate aldosterone (35). These findings might indicate that the mitochondria oxidative stress participates in the deterioration of chemical communication between heart cells. It is known that aldosterone, for instance, causes intramitochondrial Ca^2+^ overloading and oxidative stress with opening of the mitochondrial permeability transition pore (36). Although mitochondrial ROS has an important role in normal physiological cell signaling, overproduction of mitochondrial ROS elicited by high glucose as well as Ang II and hypoxia, lead to cardiovascular damage. Therefore, the possible role of mitochondrial ROS on the inhibition of intercellular glucose diffusion elicited by aldosterone cannot be discarded.

The interruption of intercellular transfer of glucose elicited by aldosterone has important consequences for tissue homeostasis and ATP synthesis. A reduction of the Na pump, for instance, results in an increment of the intracellular sodium concentration and membrane depolarization with consequent change of cardiac excitability. It is known that aldosterone generates triggered activity caused by early-afterdepolarization (EAD) and delayed afterdepolarization (DAD), which is associated with intracellular Ca^2+^ mishandling (37). Transgenic mice with cardiac-specific MR overexpression show prolonged ventricular repolarization and severe arrhythmia associated with a decrease of outward potassium current (38) and abnormal Ca^2+^ release from the sarcoplasmic reticulum during diastole with consequent generation of cardiac arrhythmias (37). Since an increase of intracellular calcium concentration is known to impair heart cell communication (14), one cannot rule out the possibility that the increment of intracellular Ca^2+^ contributes to the decrease of junctional permeability to glucose as described above.

The disruption of cell-to-cell diffusion of glucose elicited by aldosterone generates discrepant intracellular levels of glucose with consequent variation of ATP synthesis resulting in different energy levels with serious consequences for cellular function. The interruption of intercellular flow of glucose might be particularly harmful for the failing or diabetic heart in which the cardiac cell energy is already reduced and the transport of glucose through the surface cell membrane is impaired. The physiological consequences of interruption of intercellular diffusion of glucose in the heart are not known and require further studies.

### Limitations of the Present Studies

(1) Although the present measurements of oxidative stress indicated its contribution to the effect of aldosterone on the intercellular diffusion of glucose, further studies using stronger inhibitors of oxidative stress will be needed to confirm the present findings.
(2) The physiological consequences of the disruption of glucose transport through gap junction are not known. A possible result of this disruption is the generation of different intracellular levels of glucose throughout the ventricle leading to unequal intracellular ATP levels with all the consequences for cellular energetics. Certainly, further studies will be needed to clarify these points.
(3) Although previous studies revealed that aldosterone reduces the expression of Cx43 in cardiac cells of neonatal rats, similar studies will be necessary to confirm the effect of the hormone in adult-differentiated cardiomyocytes.

## Conclusion

Aldosterone disrupts the exchange of glucose between cardiac cells by reducing the gap junction permeability. The mechanism by which aldosterone changes the gap junction permeability is not known but it is probably multifactorial, including changes in cell volume, oxidative stress, and phosphorylation of gap junction proteins. Moreover, a down regulation of Cx43 (17) might contribute to the disruption of intercellular flow of glucose between the cardiomyocytes, but further studies will be needed to confirm this possibility.

## Conflict of Interest Statement

The author declares that the research was conducted in the absence of any commercial or financial relationships that could be construed as a potential conflict of interest.
